# Head and Neck Cancer Detection in Digitized Whole-Slide Histology Using Convolutional Neural Networks

**DOI:** 10.1038/s41598-019-50313-x

**Published:** 2019-10-01

**Authors:** Martin Halicek, Maysam Shahedi, James V. Little, Amy Y. Chen, Larry L. Myers, Baran D. Sumer, Baowei Fei

**Affiliations:** 1 Department of Bioengineering, University of Texas at Dallas, Richardson, TX USA; 2 Department of Biomedical Engineering, Georgia Institute of Technology and Emory University, Atlanta, GA USA; 3 Department of Pathology and Laboratory Medicine, Emory University School of Medicine, Atlanta, GA USA; 4 Department of Otolaryngology, Emory University School of Medicine, Atlanta, GA USA; 5 Department of Otolaryngology, University of Texas Southwestern Medical Center, Dallas, TX USA; 6 Advanced Imaging Research Center, University of Texas Southwestern Medical Center, Dallas, TX USA; 7 Department of Radiology, University of Texas Southwestern Medical Center, Dallas, TX USA

**Keywords:** Oral cancer detection, Cancer imaging

## Abstract

Primary management for head and neck cancers, including squamous cell carcinoma (SCC), involves surgical resection with negative cancer margins. Pathologists guide surgeons during these operations by detecting cancer in histology slides made from the excised tissue. In this study, 381 digitized, histological whole-slide images (WSI) from 156 patients with head and neck cancer were used to train, validate, and test an inception-v4 convolutional neural network. The proposed method is able to detect and localize primary head and neck SCC on WSI with an AUC of 0.916 for patients in the SCC testing group and 0.954 for patients in the thyroid carcinoma testing group. Moreover, the proposed method is able to diagnose WSI with cancer versus normal slides with an AUC of 0.944 and 0.995 for the SCC and thyroid carcinoma testing groups, respectively. For comparison, we tested the proposed, diagnostic method on an open-source dataset of WSI from sentinel lymph nodes with breast cancer metastases, CAMELYON 2016, to obtain patch-based cancer localization and slide-level cancer diagnoses. The experimental design yields a robust method with potential to help create a tool to increase efficiency and accuracy of pathologists detecting head and neck cancers in histological images.

## Introduction

Head and neck cancer is the sixth most common cancer world-wide, and majority of cancers of the upper aerodigestive tract are squamous cell carcinoma (SCC)^1^. Approximately two-thirds of SCC patients present with either stage III or IV advanced disease^2^. Surgical cancer resection remains the primary management SCC of the head and neck, with concurrent chemotherapy or radiation therapy depending on the extent of the disease^3^.

Commonly, the safe margin for surgical resection of oral squamous cell carcinoma (SCC) at sites including the surfaces of the lips, gums, mouth, plate, and anterior two-thirds of the tongue is considered 5 mm from the permanent edge of the tumor^4^. Alternative distances for resection margins have been proposed, as low as tumor clearance of 2.2 mm to be declared a “negative” margin^5^. However, closer margins, for example within 1 mm, are associated with significantly increased recurrence rates^6^. In head and neck surgical histology, there are two techniques to investigate surgical margin clearance: perpendicular sectioning and *en-face* technique. Perpendicular sectioning, also known as “bread loafing”, allows the margin clearance from the edge of the resected tissue to be easily quantified, but it is resource exhaustive and limited by the number of slices, which can create false negatives. The *en-face* technique evaluates the surface area in a longitudinal fashion to determine if there is any cancer on surface of the submitted specimen^4^. Both require examining a large quantity of histological slides and considerable diagnostic time.

Previous studies have implemented computer-assisted detection methods using histological images for machine learning^7^. Colorectal epithelial and stromal tissues have been classified on histological images using support vector machines with hand-crafted features, such as color and texture^8,9^. Additionally, convolutional neural networks (CNNs), which are a family of machine learning algorithms that learn to extract features from training images, have also been applied to classifying epithelium and stromal tissues from colorectal and breast cancers^10^. Non-small cell lung cancers, including metastatic SCC to the lungs, have been classified in histological images using CNNs that are trained to work regions of the image, called image-patches^11^. Another method for detecting lung cancers in histological images of needle core biopsies used morphological and color features for classification with an ensemble of artificial neural networks^12^. Head and neck SCC was investigated once before, but only in cell lines xenografted into mice, and a CNN was implemented with histological images to predict hypoxia of tumor-invaded microvessels^7,13^. Additionally, computerized methods have been developed for thyroid carcinomas to detect and classify malignant versus benign nuclei from thyroid nodules and carcinomas, including follicular and papillary thyroid carcinomas, in histological images on a cellular level with promising results^14–16^. However, most of the work involving thyroid carcinoma has been implemented on a cellular or nuclear level using hand-crafted features, such as texture or shape, and support-vector-machines are employed for nuclei classification, with many algorithms using an ensemble of classifiers^15–19^.

In the field of digital pathology, whole slide imaging (WSI) refers to the acquisition of high-resolution images of stained tissue slides, which retains the ability to magnify and navigate these digital slides just as standard microscopy^20^. After reviewing nearly 2,000 patient cases, it has been concluded that WSI is non-inferior to microscopy for primary diagnosis in surgical pathology across multiple staining types, specimen types, and organ systems^21^. Computer-assisted detection algorithms have recently been implemented using CNNs for diagnosis in WSI with considerable success for identifying metastasis in lymph nodes^22,23^. Several state-of-the-art methods using CNNs have been applied during a grand challenge hosted at the IEEE International Symposium for Biomedical Imaging in 2016 and 2017 to detect breast cancer metastasis in WSI of sentinel lymph nodes (CAMELYON) with AUCs up to 0.99, comparable to expert pathologists performing with an AUC of 0.81 to 0.97, with and without a time constraint^22,24,25^.

This study aims to investigate the ability of CNNs for detecting head and neck SCC and thyroid carcinomas in a novel dataset of digitized whole-slide histological images from surgical pathology. A recent literature review shows that this is the first work to investigate SCC and thyroid carcinoma detection on a WSI level in primary head and neck cancers^7^, and we implement state-of-the-art classification methods in an extensive dataset collected from our institution. The major contribution of this paper focuses on the first application of deep learning for the histological detection of H&N SCC and thyroid cancers in a sufficiently large head and neck cancer dataset that is best suited for a patch-based CNN approach. The anatomical variation of the head and neck is astonishingly complex. The inclusion of multiple, most common locations of SCC yields a successful and substantial generalization for this application. Additionally, three of the major forms of thyroid carcinoma are studied, and despite extensive morphological differences, the method allows successful performance. Altogether, the dataset and applied methodology of this work demonstrate the current potential to create a tool to increase the efficiency and accuracy of surgical pathologists performing real-time SCC cancer detection on WSI for intraoperative guidance during primary head and neck cancer resection operations.

## Materials and Methods

In this section, the materials for this study, including the cancer histological datasets, are described. Additionally, the methods of image processing, convolutional neural networks, and performance evaluation are detailed.

### Head and neck cancer dataset

Informed, written consent was obtained from all patients consented for our study. All experimental methods were approved by the Institutional Review Board (IRB) of Emory University under the Head and Neck Satellite Tissue Bank (HNSB, IRB00003208) protocol. In collaboration with the Otolaryngology Department and the Department of Pathology and Laboratory Medicine at Emory University Hospital Midtown, freshly excised, *ex-vivo* head and neck cancer tissue samples were obtained from previously consented patients undergoing surgical cancer resection^26,27^. Tissue specimens collected from patients were de-identified and coded by a clinical research coordinator before being released to our laboratory for research purposes only. Three tissue samples were collected from each patient: a sample of the tumor, a normal tissue sample, and a sample at the tumor-normal interface.

For this study, we present the first application of the histological component of this dataset of 381 WSI from 156 patients, which is detailed by dataset in Table 1. In the upper aerodigestive tract SCC group, there were 228 tissue samples collected from 97 patients. The number of patients and tissue specimens is enumerated per anatomical origin of the SCC in Table 2. The only tissues that were excluded in this study were from three patients that had SCC of Waldeyer’s ring. These tissues were excluded because they were comprised of entirely lymphoid tissue, and the samples from only 3 patients of this diverse tissue type was not sufficient for inclusion in this study. The normal specimens collected were non-dysplastic and non-cancerous, which may have inflammation, atypia, or reactive epithelium.

**Table 1:** Summary of the number of patients and whole-slide images (WSI) included in this study for training, validation, and testing of the proposed method.

Dataset	Training		Validation		Testing		Total	
	Patients	WSI	Patients	WSI	Patients	WSI	Patients	WSI
Head & Neck SCC	45	91	13	32	39	105	97	228
Thyroid Carcinoma	24	48	8	23	27	82	59	153
Breast Cancer Mets.	250	250	20	20	129	129	399	399

**Table 2:** Summary of the number of patients in the SCC dataset and WSI obtained from tissue specimens per anatomical location of the head and neck. Tissue specimens refer to the *ex-vivo* samples used to construct the histological WSI (T: tumor, N: normal, TN: tumor-normal interface).

Location	# Patients	# T	# N	# TN
Tongue	18	9	17	17
FOM	12	7	10	13
Larynx	10	10	9	3
Mucosal Gingiva	9	7	9	4
Mandible	8	6	6	5
Maxillary Sinus	6	4	6	5
Oral Cavity	6	4	7	6
Hypopharynx	5	4	5	1
RMT	5	6	3	5
Tonsil	5	4	3	6
Supraglottis	4	2	4	3
BOT	4	0	4	4
Nasal Cavity	2	2	1	0
Other	3	2	2	3
Total	97 Patients	228 WSI		

The thyroid carcinoma group was comprised of primary papillary, medullary, and follicular thyroid carcinomas. There were 153 tissue specimens collected from 59 patients, which included 47 patients with papillary thyroid carcinoma, 5 patients medullary thyroid carcinoma, and 7 patients with follicular carcinoma. Each dataset was subdivided into separate groups for training, validation, and testing of the proposed computer-assisted cancer detection algorithm.

Fresh *ex-vivo* tissues were collected from the surgical pathology department and fixed, paraffin embedded, sectioned, stained with haemotoxylin and eosin (H&E), and digitized using whole-slide scanning at an equivalent magnification to 40x objective using a NanoZoomer (Hamamatsu Photonics), which produces a final digital slide with pixel-level resolution of 0.23 μm × 0.23 μm. A board-certified pathologist with expertise in H&N pathology outlined the cancer margins on the digital slides using Aperio ImageScope (Leica Biosystems Inc, Buffalo Grove, IL, USA).

### Breast cancer lymph node metastases dataset

For external validation, we implemented the proposed cancer detection algorithm on the open-source CAMELYON 2016 dataset^23,28^, in order to compare the results of our proprietary head and neck cancer dataset since currently no similar independent dataset exists. The CAMELYON 2016 dataset consists of 399 whole-slide digital images from sentinel lymph nodes (SLN) obtained from 399 patients, one SLN from each patient that underwent breast cancer surgical resection. The dataset is collected at two institutions: Radboud University Medical Center (RUMC) Netherlands and University Medical Center Utrecht (UMCU) Netherlands^23,28^. One slide was constructed from one SLN from each patient. Table 1 shows the numbers of patients and slides in each group.

The whole-slide images were digitized at each institution separately, so the different hospitals each use a different scanner. The slides that were digitally scanned at RUMC were produced at 20x objective magnification using a Pannoramic 250 Flash II digital slide scanner (3DHISTECH), which corresponds to the pixel-level resolution of 0.24 μm × 0.24 μm. The slides that were digitized at UMCU were acquired with a NanoZoomer-XR digital slide scanner at 40x objective magnification (Hamamatsu Photonics) with a pixel-level resolution of specimens of 0.23 μm × 0.23 μm^23,28^.

### Histological image processing

The histological dataset presented consists of primary tumor specimens acquired from surgical resections. Our SCC and thyroid cancer datasets do not have fine cellular-level annotations. Instead, regions were broadly marked as cancer if there were any cancer cells present, even if surrounded by normal structures, to establish which areas would require surgical removal. For this task, cell-by-cell annotations are not necessary. Clinicians require accurate regional diagnosis of cancer invaded tissues with an estimate of border clearance distance to the edge of the resected tissue. Therefore, the nature of the ground-truth for this work necessitates a patch-based deep learning approach. Moreover, a fully-convolutional network (FCN), as is widely used in the literature, would be problematic for this approach. Firstly, the tissue specimens of primary cancers collected tend to have large regions of each class. Therefore, the large majority of patches tend to be one class (all normal or all tumor), with few border patches that contain both classes. This would create problems with loss calculation and gradient optimization for training an FCN. Lastly, as stated the ground truth is coarse, so if a FCN could be adequately trained to produce fine-level segmentations, not only are they not needed for this task, but the ground truth would call potentially correct areas as misclassifications.

**Figure 1:**
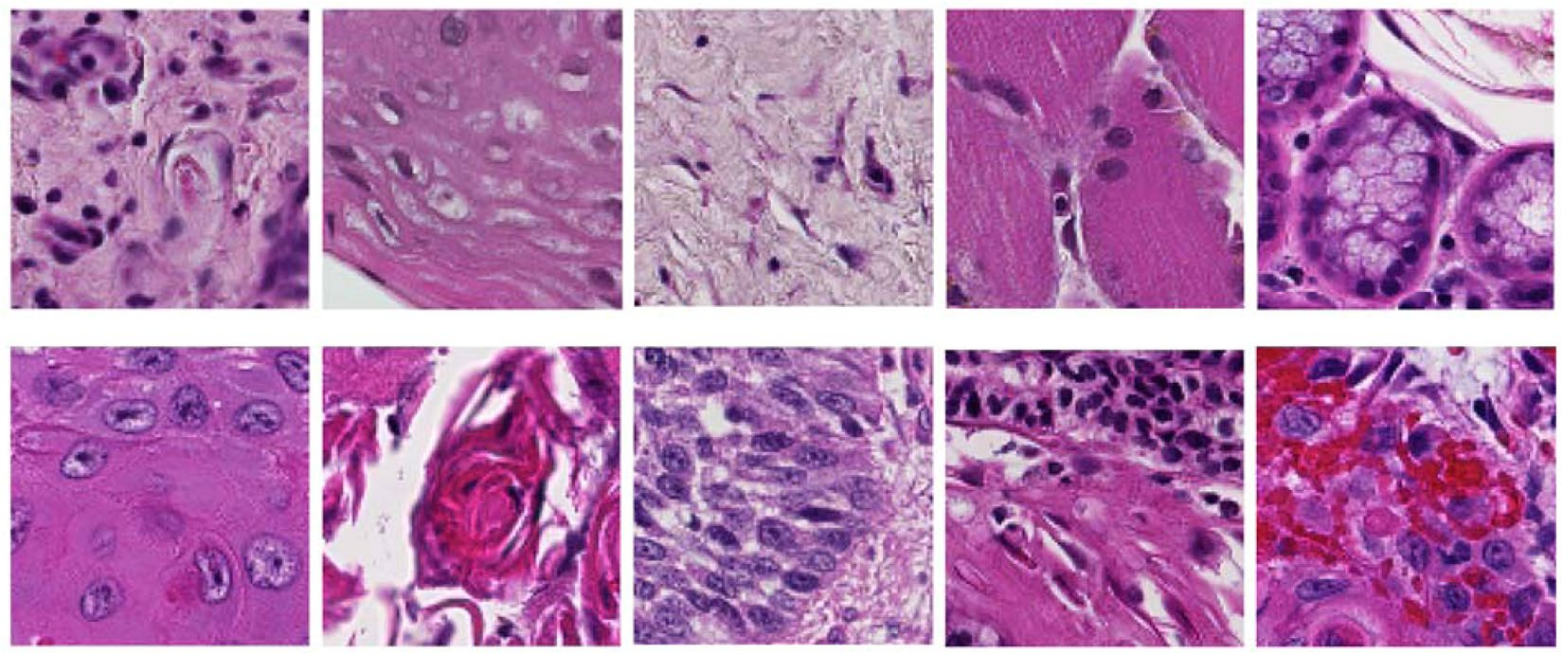
Histological images (101 × 101 pixel image-patches) showing anatomical diversity. Top: Patches of various normal structures, including chronic inflammation, stratified squamous epithelium, stroma, skeletal muscle, and salivary glands (from left to right). Bottom: Patches of SCC with varying histologic features: keratinizing SCC, keratinizing SCC with keratin pearls, basaloid SCC, SCC with chronic inflammation, SCC with hemorrhage (from left to right).

A ground-truth binary mask of the cancer area is produced from each outlined histology slide. The WSIs and corresponding ground-truths were down-sampled by a factor of four using nearest neighbor interpolation. The proposed method classifies the WSI in a patch-based method using a window that slides over the entire image. Due to the unique challenges of working with digital pathology images, which can create datasets of hundreds of images that are each tens of gigabytes, it is the current state-of-the-art to perform both down-sampling and patch-based image reconstruction approaches to computationally handle this type of data^22,29–35^. Image patches (***I***) are produced from each down-sampled H&E slide using 101 × 101 pixels and are labeled corresponding to the center pixel, where ${\boldsymbol{I}}\in {{\mathbb{R}}}^{101\times 101\times 3}$. Representative patches from H&N SCC are shown in Fig. 1 showing the histological variation of normal anatomical structures and various appearances of SCC of various identifiable difficulty. The SCC and thyroid carcinoma training groups were comprised of patches only from the tumor and normal tissue WSI, and the validation and testing groups were comprised of patches from all slides. Since the lymph node dataset contained more WSI but with smaller cancer areas, the training dataset was constructed by taking up to 5000 image patches from the cancerous area of each of the 101 cancer WSI in the training dataset, and using up to 1000 image patches from each slide of the 149 normal WSI. The training group was approximately balanced between cancer and normal patches for better performance.

Histology slides have no canonical orientation, meaning the tissue will have the same diagnosis from all vantage points. Therefore, the number of image patches were augmented by 8x by applying 90-degree rotations and reflections to develop a more robust diagnostic method. Additionally, to establish a level of color-feature invariance and tolerance to differences in H&E staining between slides, the hue, saturation, brightness, and contrast of each patch were randomly manipulated to make a more rigorous training paradigm.

### Convolutional neural network implementation

The three distinct cancer datasets in this study were employed to separately train, validate, and test a 2D-CNN classifier based on the Inception V4 architecture, implemented in TensorFlow on 8 Titan-XP NVIDIA GPUs^36–39^. The Inception V4 CNN architecture was modified slightly in the early layers, which is detailed in Table 3, in order to accommodate the patch-size selected for this study. The CNN architecture consisted of 3 convolutional layers and 1 max-pooling layer to accommodate the patch-size used, and in total the CNN contained 141 convolutional layers and 18 pooling layers^37,39^. Gradient optimization was performed using the Adadelta optimizer with an initial learning rate of 1.0 that was exponentially decayed by 0.95 every 3 epochs of training data^40^. The softmax cross entropy was used as the loss function. If the *k*^*th*^ training patch is denoted as ${{\boldsymbol{I}}}_{k}\in {{\mathbb{R}}}^{101\times 101\times 3}$, $k=1,\,2,\ldots ,\,K$, where *K* is the number of training patches in a batch, the CNN training process is to find a function $F:{{\mathbb{R}}}^{101\times 101\times 3}\to {\mathbb{R}}$ that minimize the following cost function ${\mathcal L}$:

$$ {\mathcal L} =-\,\frac{1}{K}\mathop{\sum }\limits_{k=1}^{K}[{g}_{k}^{N}log({p}_{k})+{g}_{k}^{P}log(1-{p}_{k})]$$

where

$${p}_{k}=\frac{{e}^{F({{\boldsymbol{I}}}_{k})}}{{\sum }_{j=1}^{K}{e}^{F({{\boldsymbol{I}}}_{j})}}$$

and ${g}_{k}^{N}$ and ${g}_{k}^{P}$ are the ground truth labels for cancer-negative and cancer-positive tissue classes, respectively, corresponding to the *k*^*th*^ patch.

**Table 3:** Schematic of the proposed modified Inception V4 CNN. The input size is given in each row, and the output size is the input size of the next row. All convolutions were performed with sigmoid activation and 40% dropout.

Layer	Kernel size/Remarks	Input Size
Conv	3 × 3/‘valid’	101 × 101 × 3
Conv	3 × 3/‘valid’	98 × 98 × 32
Max Pool	2 × 2/stride=2 ‘valid’	96 × 96 × 64
Conv	3 × 3/stride=2 ‘valid’	48 × 48 × 64
4 x Inception-A Block	1 × 1 and 3 × 3/‘same’	23 × 23 × 80
Reduction-A Block	1 × 1 and 3 × 3/‘same’	23 × 23 × 384
7 × Inception Block	1 × 1, 1 × 7, 7 × 1, and 3 × 3/‘same’	11 × 11 × 1024
Reduction-B Block	1 × 1, 1 × 7, 7 × 1, and 3 × 3/‘same’	11 × 11×1024
3 × Inception-C Block	1 × 1, 1 × 3, 3 × 1, and 3 × 3/‘same’	5 × 5 × 1024
Avg. Pool	5 × 5/‘valid’	5 × 5 × 1536
Linear	Logits	1 × 1536
Softmax	Classifier	1 × 2

The validation groups were used to determine the optimal number of training epochs used for each of the three datasets. Each CNN was trained with a batch size of 128 image patches, and batches were converted from RGB to HSV before being passed into the CNN. Both RGB and HSV were tested in early validation experiments, and HSV without any other modification out-performed RGB results. One reason could be the separation of the image intensity from the color information in HSV color model. Additionally, one major challenge of H&E stained images is inconsistency of the stain quality. To demonstrate that color feature augmentation can solve this problem, working in HSV directly, the hue, saturation, and brightness were perturbed randomly in each channel independently. The SCC CNN was trained for 30 epochs of training data, equivalent to 295,000 steps using a batch-size of 128 patches. The random color augmentation was using the native color feature variance in the training group: hue 4%, saturation 15%, brightness 8%, and contrast 2%. The thyroid carcinoma CNN was trained for 70 epochs of training data (equivalent to 433,000 steps). HSV and contrast perturbation was 5%, 5%, 8%, and 5% respectively. The breast cancer SLN metastasis CNN was trained for 20 epochs (equivalent to 203,400 steps). HSV and contrast were each randomly perturbed in range of −10% to 10%.

### Image reconstruction and post processing

Each of the *N* testing slides (${{\boldsymbol{S}}}^{{T}_{t}},\,t=1,2,\ldots ,N$) were tiled into image patches of size 101 × 101 pixels (${{\boldsymbol{I}}}_{m,n}^{{T}_{t}}$), produced with a stride of 50 pixels, which makes an overlap of 51 pixels.

$${{\boldsymbol{I}}}_{m,n}^{{T}_{t}}=\{{{\boldsymbol{S}}}^{{T}_{t}}(x,y)|m\le x\le m+100,n\le y\le n+100\}$$

where $m=1,51,101,\ldots ,{M}_{t}$, $n=1,51,101,\ldots ,{N}_{t}$, and *M*_*t*_ × *N*_*t*_ is the size of the *t*^*th*^ testing slide. Therefore, the final classified whole-slide image is a cancer probability heat-map with a level of detail equal to 50 × 50 pixel blocks. Each image patch, ${{\boldsymbol{I}}}_{m,n}^{{T}_{t}}\in {{\mathbb{R}}}^{101\times 101\times 3}$, was classified in all 8 orientations with randomized HSV and contrast features and averaged to obtain a single cancer prediction value, according to the following equation.

$$\bar{p}({{\boldsymbol{I}}}_{m,n}^{{T}_{t}})=\frac{1}{8}\mathop{\sum }\limits_{s=1}^{8}{p}_{s}({{\boldsymbol{I}}}_{m,n}^{{T}_{t}})$$

where ${p}_{s}({{\boldsymbol{I}}}_{m,n}^{{T}_{t}})$ is the cancer prediction for the *s*^*th*^ orientation of ${{\boldsymbol{I}}}_{m,n}^{{T}_{t}}$ and $\bar{p}({{\boldsymbol{I}}}_{m,n}^{{T}_{t}})\in [0,1]$ is the average cancer prediction of the patch. Additionally, the results of overlapping image patches were averaged in the overlapping area, as follows.

$$\bar{q}({{\boldsymbol{H}}}_{i,j})=\frac{1}{4}(\bar{p}({{\boldsymbol{I}}}_{i,j}^{{T}_{t}})+\bar{p}({{\boldsymbol{I}}}_{i+49,j}^{{T}_{t}})+\bar{p}({{\boldsymbol{I}}}_{i,j+49}^{{T}_{t}})+\bar{p}({{\boldsymbol{I}}}_{i+49,j+49}^{{T}_{t}}))$$

where $\bar{q}$ is the final probability of the final resolution block size (${{\boldsymbol{H}}}_{i,j}\in {{\mathbb{R}}}^{50\times 50}$) of the heat-map. The benefit of this post-processing method was to increase the resolution of the heat-map from 101-pixel image patches to 50-pixel image patches. Moreover, the image patches that constituted the free edge of the tissue were averaged less than four times because they did not have the complete number of neighboring patches. This image reconstruction and post-processing method was determined to increase accuracy by about 2% in early validation experiments.

To investigate the ability of the CNN to detect cancer on histological images, we implemented the gradient class-activated map (grad-CAM) method to visualize gradients activated by each class for the example input image patches^41^. We traced the gradients from the last convolutional layer before the inception modules to the logits layer to separately visualize cancer and normal components. This technique produces a weighted combination of the convolutional filters and gradients as the CNN is activated by a specific input image for each class.

### Performance evaluation

The reference standard cancer margin was annotated by hand for all digital slides employed in this study. For the head and neck cancer database, a board-certified pathologist with expertise in H&N pathology outlined the cancer margins on the digital slides. For the breast cancer metastasis database, an experienced lab technician and a clinical Ph.D. student outlined the cancer margins, which were then confirmed by one of two board-certified pathologists with expertise in breast cancer^28^.

During training, the performance of the validation group was calculated and monitored. The optimal operating threshold was calculated from the validation group for generalizable results, and it was used for generating performance evaluation metrics for the testing group. To reduce bias in the experiment, the fully-independent testing group was only classified a single time at the end of the experiment, after all the network optimizing had been determined using the validation set. To test the ability to diagnose and localize cancer on WSI, we used AUC, F1 score, accuracy, sensitivity, and specificity to evaluate cancer detection on a patch-based level. Confidence intervals were calculated using a boot-strapping algorithm. Additionally, the ability of the proposed algorithm to diagnose slides with cancer from normal slides was investigated. This slide-level AUC was calculated by assigning the value of the image patch with the maximum cancer probability to the entire WSI.

### Informed consent

Informed written consent was obtained from all patients prior to participation in this study. All methods were carried out in accordance with the approved Institutional Review Board protocols and the relevant guidelines and regulations of Emory University.

## Results

Head and neck primary SCC was detected on digitized WSI with an AUC of 0.916 and 85% accuracy for patients in the testing group. The ideal threshold for distinguishing SCC from normal tissue was SCC probability of greater than 62%. Thyroid carcinoma was detected on digitized WSI with an AUC of 0.954 and 89% accuracy for patients in the testing group. The ideal threshold for distinguishing thyroid carcinoma from normal thyroid tissue was cancer probability of greater than 50%. Breast cancer lymph node metastasis was detected on digitized WSI with an AUC of 0.967 and 93% accuracy for patients in the testing group. The ideal threshold for identifying metastasis in SLNs was cancer probability of greater than 28%. Reported in Table 4 are the AUC for the validation groups and the AUC, accuracy, sensitivity, and specificity of the testing groups.

**Table 4:** Cancer detection results, obtained from ROC curves using all histological images’ patch-level statistics. Reported are the AUC for the validation group and the AUC, F1 score, accuracy, sensitivity, and specificity of the testing group with 95% confidence intervals for all values. Also shown in the right-most column is the ability of the proposed method to distinguish slides that contain cancer from slides that are all normal.

Group	Validation AUC	Test AUC	F1 Score	Accuracy	Sensitivity	Specificity	Slide Level AUC
SCC	0.913 (0.90,0.93)	0.916 (0.90, 0.93)	84.8 ± 1.5%	84.8 ± 1.6%	84.7 ± 2.2%	85.0 ± 2.2%	0.944 (0.91, 0.97)
Thyroid	0.927 (0.92, 0.94)	0.954 (0.94, 0.97)	89.4 ± 1.3%	89.4 ± 1.3%	89.6 ± 1.8%	89.1 ± 1.9%	0.995 (0.99, 1.00)
Lymph Node	0.986 (0.96, 0.99)	0.967 (0.96, 0.98)	91.8 ± 1.3%	93.4 ± 1.2%	90.1 ± 1.8%	93.6 ± 1.6%	0.901 (0.86, 0.94)

**Figure 2:**
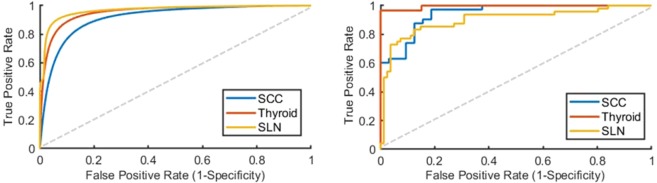
The ROC curves are shown for patch-level cancer detection (left) and slide-level cancer diagnosis (right) in the testing groups from all three datasets. The dotted gray line corresponds to random guess.

**Figure 3:**
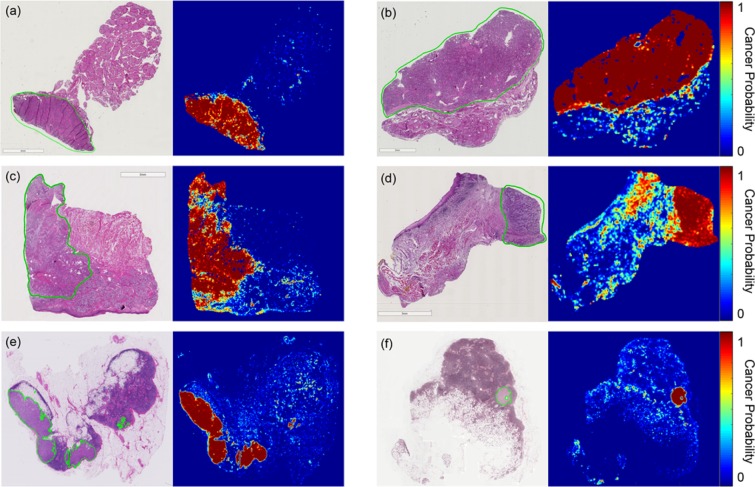
Representative whole-slide classification results. (**a**,**b**) Papillary thyroid carcinoma WSI from two patients. (**c**,**d**) SCC WSI from patients with tongue and retromolar trigone SCC. (**e**,**f**) Breast cancer metastasis to lymph node WSI from two patients. The cancer area is outlined in green on the H&E images, and the heat maps are shown of the cancer probability.

**Figure 4:**
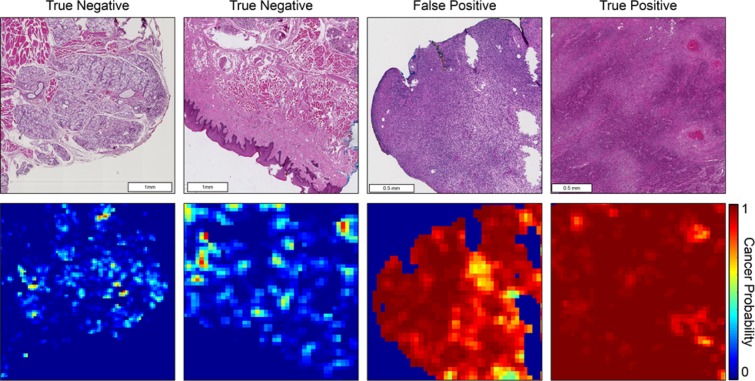
Heat maps representing cancer probability of several regions of interest. From left to right, the CNN correctly identifies salivary gland and muscular components as having a low probability of SCC; stratified squamous epithelium correctly shown as a true negative; a false positive area representing inflammatory infiltration near the SCC border (not shown); correctly classified true positive SCC classified with a high probability of SCC.

**Figure 5:**
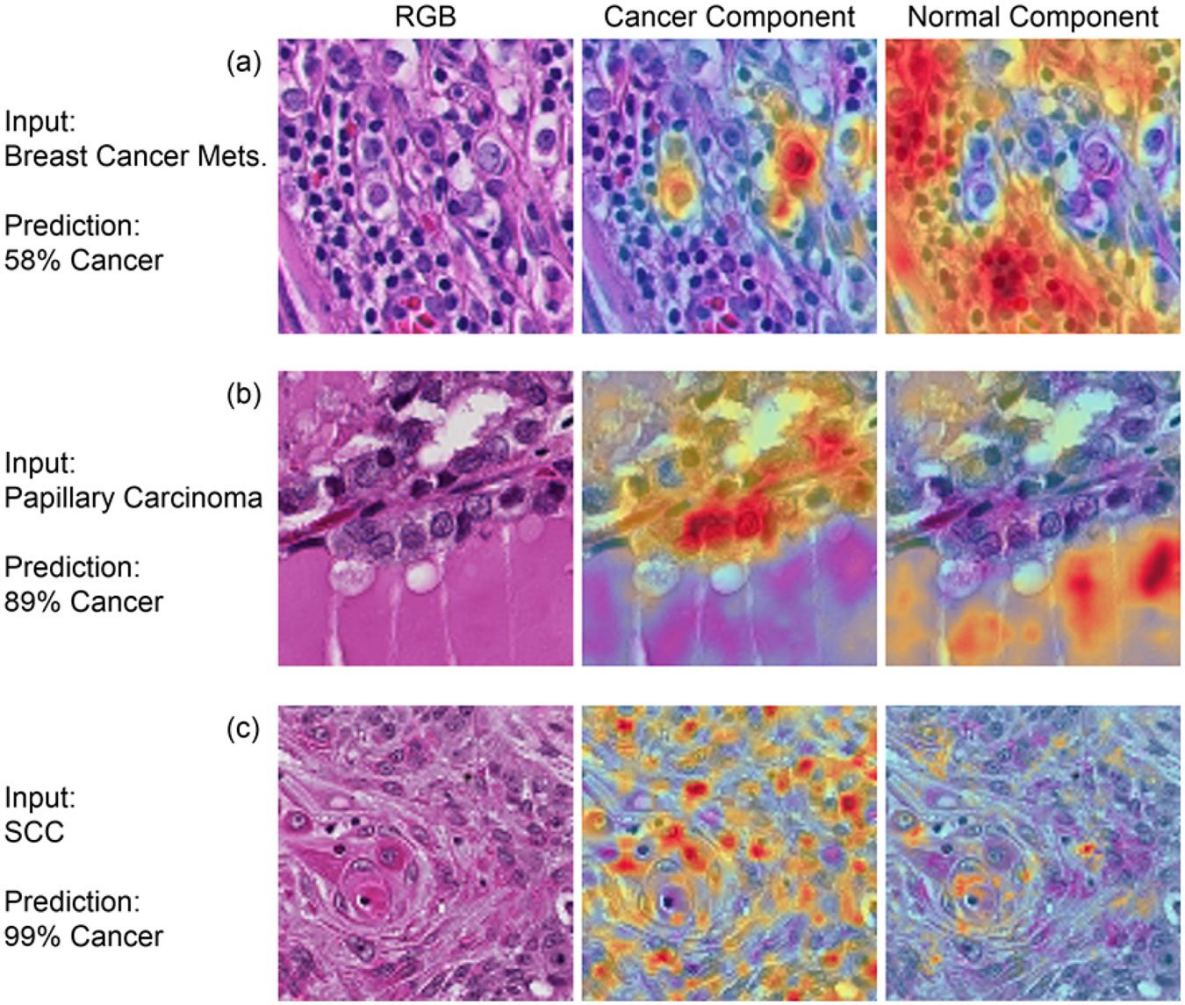
Representative, correctly-classified cancer image-patches from each dataset, visualized with cancer and normal components using the grad-CAM technique^41^. The gradients were traced from the last convolutional layer before the inception modules to the cancer and normal logits separately to visualize contributions from cancer and normal features leading to a correct cancer prediction. (**a**) Breast cancer SLN metastasis image patch correctly classified as cancer with 58% probability. (**b**) Papillary thyroid carcinoma image patch correctly classified as cancer with 89% probability. (**c**) SCC image patch correctly classified as cancer with 99% probability.

Receiver operator characteristic (ROC) curves for slide-level and patch-level cancer detection in the testing groups from all three datasets are shown in Fig. 2. Patch-level ROC curves are generated using all histological images’ patch-level data for cancer localization, and slide-level ROC curves demonstrate WSI diagnosis. Additionally, two representative WSI from each of the three testing groups and their corresponding predicted heat-maps are shown in Fig. 3. Several regions of interest (ROI) are detailed in Fig. 4 to identify the strengths and weaknesses of the proposed method in the detection of SCC. The ideal threshold for whole-slide level detection of SCC was above 95% probability, so the regions detailed as true negatives in Fig. 4 fall below this threshold. Additionally, the grad-CAM technique was used to visualize the contributing normal and cancerous components of a few example input images that were corrected classified with high probability (Fig. 5). This approach reveals that a contribution of the cancer prediction is made by nuclear features.

The ability of the proposed method to diagnose the entire WSI that contain any cancer was also investigated. WSIs with SCC were diagnosed with an AUC of 0.944. Thyroid carcinoma WSIs were diagnosed with an AUC of 0.995. WSI of SLN with breast cancer metastases were diagnosed with an AUC of 0.901.

## Discussion

In this work, we present a new and extensive histological dataset of primary head and neck cancer and implement a state of the art Inception V4 CNN architecture for cancer detection and WSI diagnosis. The results are generalizable because of the division of patients across training, validation, and testing. To the best of our knowledge, this is the first work to investigate SCC detection in digitized whole-slide histological images from primary head and neck cancers.

The digitized, whole-slide histological images were saved as TIF files with resolution equivalent to 40x microscopic objective. After 4x down-sampling, the image patches correspond to 10x objective equivalence. Different down-sampling factors and patch-sizes were explored, but this method yielded the best validation group results, so it was used for testing. Similarly, pathologists detecting SCC in histology slides use a variety of objectives, not exclusively 40x, which may be too zoomed-in to determine if the region is cancerous or benign. We see this issue in our dataset as well. It is not only possible, but likely that in some slides labeled as ‘tumor only’, there may be some areas inside the tissue, or in between tumor nests, that is entirely normal. Therefore, it is understandable that classification using 4x down-sampled images obtains the highest accuracy. Other CNN architectures were explored in early experiments using the validation set only, and various patch-sizes were experimented with, but ultimately the Inception V4 CNN architecture with a patch-size of 101 × 101 × 3 in HSV color space, yielded the most promising validation results.

Additionally, the regions of interest that are presented show true negative, false positive, and true positive regions that vary from 1 to 3 mm in size. These results demonstrate the proposed method is able to distinguish normal anatomical structures like epithelium and salivary gland from SCC with high probability. Also, the most common false positive observed in the classified result is tissue areas that contain dense inflammation. This result is most likely a by-product of the training paradigm. As SCC develops, there is an accompanying immune response that leads to massive inflammatory infiltration into the tissue^42^. Therefore, the proposed algorithm learned the association between SCC and inflammation.

To our knowledge, there are no other studies that attempt to detect or diagnose H&N SCC or thyroid carcinoma on WSI, and we used a proprietary dataset collected from patients at our institution. Therefore, we wanted to test the proposed, diagnostic algorithm on a similar, open-source dataset for comparison. Our slide-level results would have placed 3^rd^ in the original CAMELYON 2016^23,28^.

The grad-CAM technique was used to visualize what components of the input image are determined as useful features with a significant contribution to the cancer prediction from the CNN, as shown in Fig. 5. This reveals that the decision is made by looking at the nuclei, just like a pathologist detects cancer. The proposed method does not segment all cancerous nuclei in the image patch, but it identifies a few cancerous nuclei with a high probability of being cancer and uses this information for making the decision. We did not train the proposed algorithm specifically with this in mind. Rather, this phenomenon was learned naturally by the training paradigm. The trained CNN model also has a level of stain invariance.

## Limitations

One limitation of the presented approach results from the application of the down-sampled resolution and the patch-based Inception V4 CNN implementation. After down-sampling, each pixel represents approximately 0.91 microns, which produces a patch size that spans about 92 microns in each x-y dimension for the patch size of 101 × 101 that was implemented in this approach. The typical diameter of an SCC single-cell nucleus in our dataset was about 12 microns, which agrees with values reported in the literature of about 13 +/−2 microns^43^. Therefore, the theoretical limit of the smallest carcinoma that could be detected would be a nest of SCC cells with an approximate diameter of 92 microns. This value corresponds with an SCC nest on the order of tens of cells of SCC, depending on the cytoplasmic overlap in the arrangement of the SCC nest.

**Figure 6:**
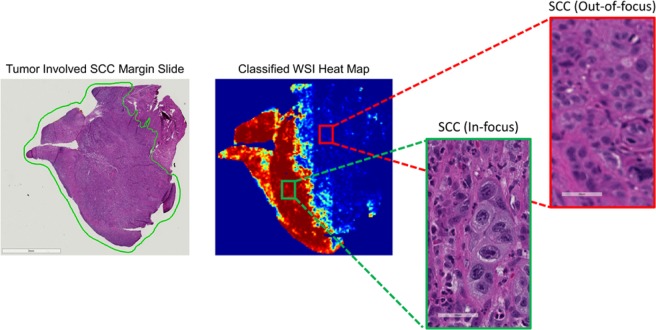
Representative false negative resulting from slide scanning artifacts. This accounts for the misclassification result of the lowest performing WSI in the SCC test group. The left side of the WSI was a correct true positive classification (green outlined box), but the out of focus artifact results in false negative misclassification (red outlined box).

Another limitation of this approach was that the algorithm suffered from whole-slide scanning artifacts, such as out-of-focus regions and including errors from slide processing, such as tissue folding and tearing. This was discovered after the completion of the experiment, and the effect was substantial, accounting for the reason in misclassification of the lowest performing WSI in the SCC testing dataset, which is shown in Fig. 6. As can been in Fig. 6, the left side of the WSI is classified correctly as a true positive SCC region, but the out-of-focus regions result in misclassification a similar ROI (shown in the cut-out boxes on the right) to be classified as false negative incorrect result. These misclassifications were retained in the testing dataset to not manipulate or bias the results, but in future work, slide scanning artifact detection should be additionally performed to determine which slides cannot be classified because of limited quality.

Digital pathology with WSI allows pathologists to view high-resolution histological images, just as standard microscopy, and it was concluded that digital pathology is non-inferior to microscopy for primary diagnosis in surgical pathology cases across multiple institutions, staining types, and organ systems^20,21^. Therefore, we believe the robust experimental procedure of the proposed method, designed to eliminate bias, has demonstrated potential benefit in a modern, digitized clinical setting. However, primary diagnosis of surgical specimens for intraoperative guidance is performed on frozen-sections rather than formalin-fixed, paraffin embedded tissues, as were investigated in this study. Additionally, frozen-sections are typically lower quality than those created from fixed, embedded specimens because they suffer from many different artifacts and depend heavily on the skill of the operator. Therefore, we believe the presented work demonstrates potential for clinical benefit, but more investigation needs to be performed. Moreover, the generalization of the results beyond head and neck cancers to breast cancer metastasis in sentinel lymph nodes suggests this method is not limited to any organ system and could be adapted to serve multiple purposes if implemented in a more clinical setting.

## Conclusion

In summary, this work focuses on the first application of deep learning for the histological detection of H&N SCC and thyroid cancers. The proposed method is able to detect and localize primary head and neck SCC on whole-slide, digitized histological images with an AUC of 0.916 for patients in the SCC testing group and 0.954 for patients in the thyroid carcinoma testing group. Moreover, the proposed method is able to discriminate WSI with cancer versus normal slides with an AUC of 0.944 and 0.995 for the SCC and thyroid carcinoma testing groups, respectively. The SCC detection method is performed across all anatomical locations, which indicates the algorithm is not limited to one location of the head and neck anatomy. For thyroid cancers, three major thyroid carcinoma are studied together which additionally demonstrates the generalizability of the method. For external validation, we tested the proposed method on an open-source dataset, CAMELYON 2016, and obtained good results. The agreement between validation and testing demonstrate that the technique is generalizable due to the robustness of the training paradigm and the careful experimental design to reduce bias. Together, the novel application to our dataset and promising results of this work demonstrate potential that such methods as the one proposed could help create a tool to increase efficiency and accuracy of pathologists performing head and neck cancer detection on histological slides for intraoperative guidance during head and neck cancer resection operations.
